# Association between brachial-ankle pulse wave velocity and progression of coronary artery calcium: a prospective cohort study

**DOI:** 10.1186/s12933-015-0311-3

**Published:** 2015-11-04

**Authors:** Jong-Young Lee, Seungho Ryu, Sung Ho Lee, Byung Jin Kim, Bum-Soo Kim, Jin-Ho Kang, Eun Sun Cheong, Jang-Young Kim, Jeong Bae Park, Ki-Chul Sung

**Affiliations:** Division of Cardiology, Department of Medicine, Kangbuk Samsung Hospital, Sungkyunkwan University School of Medicine, #108, Pyung Dong, Jongro-Ku, Seoul, 110-746 Republic of Korea; Department of Occupational and Environmental Medicine, Sungkyunkwan University School of Medicine, Seoul, Republic of Korea; Department of Cardiology, Wonju College of Medicine, Yonsei University, Wonju, Republic of Korea; Department of Medicine/Cardiology, Cheil General Hospital, Dankook University College of Medicine, Seoul, Republic of Korea

**Keywords:** Arterial stiffness, baPWV, Cardiometabolic risk factors, Cardiovascular disease (CVD), Coronary arterial calcification

## Abstract

**Background:**

Few studies have investigated the association between coronary artery calcium (CAC) progression and arterial stiffness measured by brachial-ankle pulse wave velocity (baPWV). We examined the influence of the severity of baseline baPWV on CAC progression in a large prospective cohort.

**Methods:**

A total of 1600 subjects who voluntarily participated in a comprehensive health-screening program between March 2010 and December 2013 and had baseline baPWV as well as CAC on baseline and serial follow-up computed tomography performed approximately 2.7 ± 0.5 years apart were enrolled in the study.

**Results:**

A total of 1124 subjects were included in the analysis (1067 men; mean age, 43.6 ± 5.1 years). An increased CAC score was found in 318 subjects (28.3 %) during the follow-up period. Baseline higher baPWV was significantly correlated with CAC progression, especially in subjects with third- and fourth-quartile values (adjusted odds ratio [OR] 2.04; 95 % confidence interval [CI] 1.33–3.15 and OR 2.14; 95 % CI 1.34–3.41, respectively) compared with the lowest-quartile values (P for trend <0.001). A similar effect was observed in diabetic subjects. Among the 835 subjects with a baseline CAC score = 0, progression to CAC score >0 was associated with male sex, diabetes, and higher baPWV. However, among the 289 individuals with a baseline CAC score >0, only the presence of CAC itself was predictive of CAC progression.

**Conclusions:**

Higher arterial stiffness measured by baPWV could be significantly associated with CAC progression.

**Electronic supplementary material:**

The online version of this article (doi:10.1186/s12933-015-0311-3) contains supplementary material, which is available to authorized users.

## Background

The presence of coronary artery calcium (CAC) is significantly correlated with the risk of coronary heart disease [1] and poor prognosis [2–6]. CAC can be measured using either electron beam computed tomography (CT) or multidetector CT (MDCT) by the Agatston scoring method [7]. In both men and women, CT-measured CAC values are highly sensitive for the presence of ≥50 % angiographic stenosis but only moderately specific (91 and 49 %, respectively) [8]. However, the absence of CAC is highly predictive of the absence of significant coronary artery stenosis and is used to identify individuals at low clinical risk [9].

Although serial measurements of CAC may be useful to assess the activity of the atherosclerotic process or monitor the efficacy of medications used to slow or halt the progression of coronary atherosclerosis, serial CAC studies are of unproven clinical value and are thus not recommended. However, several studies have shown an association between CAC progression and traditional risk factors and increased risk of cardiovascular events [10–12].

Arterial stiffness measured by aortic pulse wave velocity (PWV) is a potential predictor of cardiovascular events [13, 14]. Brachial-ankle pulse wave velocity (baPWV), a promising technique used to assess arterial stiffness, is simple, noninvasive, and nonradiating. BaPWV may provide information similar to that derived from central PWV [15] and is independently associated with the presence and severity of coronary artery disease (CAD) [16–18].

Because few studies have investigated the association between baPWV and CAC progression, it is unclear whether baPWV can be used to identify subjects with progressive subclinical CAC. We examined the influence of the severity of baseline baPWV on CAC progression in a prospective cohort.

## Methods

### Study subjects

**Fig. 1:**
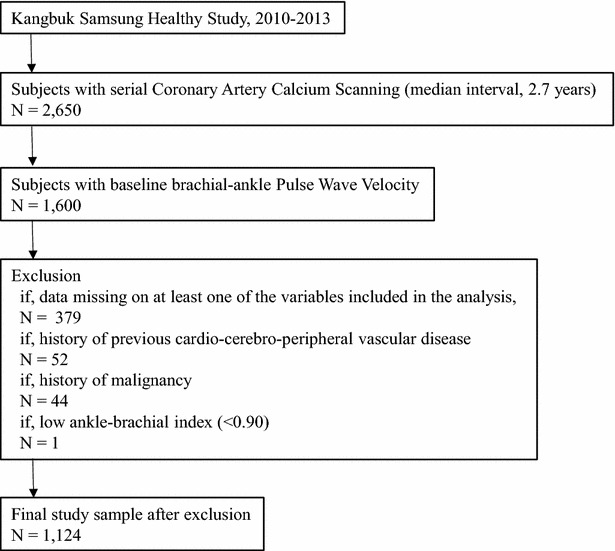
Study population. This flowchart summarizes the source and study population of this study

The Kangbuk Samsung Health Study (KSHS) is a Korean cohort study enrolling all men and women 18 years of age or older who voluntarily undergo comprehensive health-screening examinations at the Kangbuk Samsung Hospital Healthcare Centers in Seoul and Suwon, South Korea. Our study included all KSHS participants who had undergone cardiac CT scanning and baPWV measurements as part of their chosen comprehensive health-screening examination from March 2010 to December 2013 (Fig. 1). A total of 2650 subjects had CAC on baseline and serial follow-up scans performed approximately 2.7 ± 0.5 years apart. Among them, 1,600 subjects underwent baseline baPWV during the same period as the first CT scan. We excluded 97 subjects with a history of definite cardio-cerebro-peripheral vascular disease (n = 52), low ankle-brachial index (ABI) (n = 1), or malignancy (n = 44). An additional 379 subjects were excluded because of missing data for the variables included in this study. Finally, total 1124 subjects were included in this analysis (1067 men and 57 women; mean age, 43.6 ± 5.1 years). This study was approved by the Institutional Review Board of the Kangbuk Samsung Hospital.

### Data collection

All examinations were conducted at the Kangbuk Samsung Health Screening Center clinics in Seoul and Suwon by trained personnel, following a standardized protocol. A self-administered questionnaire was used to collect information about sociodemographic characteristics, lifestyle factors, medical history, family history, and medication use. Body mass index (BMI) was calculated as weight in kilograms divided by height in meters squared. Trained examiners measured blood pressure in the sitting position at least three times using an automated oscillometric device. Blood was drawn from participants after fasting for ≥10 h and analyzed at the Laboratory Medicine Department at the Kangbuk Samsung Hospital. Diabetes was defined as a self-reported history of diabetes, the use of glucose-lowering medications, and/or HbA1c ≥6.5 %.

BaPWV was recorded in the supine position using a VP-1000 (OMRON, Kyoto, Japan), which measures bilateral brachial and posterior tibial artery pressure waveforms using an oscillometric method with cuffs placed on both arms and ankles. BaPWV was calculated automatically for each arterial segment as the path length divided by the corresponding time interval. A validation study of baPWV measurements in an Asian population reported interobserver and intraobserver correlation coefficients of 0.98 and 0.87, respectively [19]. The coefficients of variation in our sample for left and right baPWV were 12.3 and 12.6 %, respectively. The average baPWV was adopted as the mean of the right and left measurements and used for the analyses.

CT scans were obtained using a Lightspeed VCT XTe-64 slice MDCT scanner (GE Healthcare, CA, USA). All scans used the same standard scanning protocol (2.5 mm slice thickness, 400 ms rotation time, 120 kV tube voltage, and 124 mAS ECG-gated dose-modulated tube current (310 mA × 0.4 s)). CAC was scored following the standard Agatston method [20]. CAC measurements had interobserver and intra-observer intraclass correlation coefficients of 0.99 under this protocol. CAC values were scored by a certified CT technologist and were subsequently blindly overread by a board-certified at least two, radiologist with sufficient experience.

### Statistical analysis

Demographic characteristics and cardiovascular risk factors of the study participants were calculated and stratified by baPWV quartiles. Categorical variables are presented as number (%) and continuous variables as mean (standard deviation [SD]) or median (interquartile range [IQR]) based on the distribution of data. Differences across quartiles were tested using Chi square or analysis of variance (ANOVA) tests. The distribution of continuous variables was evaluated, and right-skewed variables (triglycerides, alanine transaminase [ALT], gamma-glutamyltransferase [GTP], and high-sensitivity C-reactive protein [hsCRP]) were log transformed for the one-way ANOVA. To test for linear trends, we included the median value of each category as a continuous variable in the regression model. To evaluate the association of CAC progression across baPWV quartiles, we used a binomial logistic regression model to estimate odds ratios (ORs) with 95 % confidence intervals (CIs) for CAC progression. We used three models to progressively adjust for potential confounders. We initially adjusted for age. Model 1 was further adjusted for sex, center, year of screening examination, smoking status, alcohol intake, educational level, BMI, diabetes, hypertension, and high-density lipoprotein (HDL), low-density lipoprotein (LDL), and glucose levels. In model 2, we further adjusted for systolic blood pressure and heart rate. Finally, we analyzed the impact of baPWV and pulse pressure (PP) on CAC progression by dividing subjects into two groups according to median baPWV and PP values.

A two-sided P value <0.05 was considered significant. Statistical analyses were performed using Stata version 12 (Stata Corp. 2011, College Station, TX, USA).

## Results

A total of 1124 subjects had CAC on baseline and follow-up scans performed approximately 2.7 ± 0.5 years apart. Their mean age was 43.6 years, and 94.9 % were men; 31.8 % of subjects were current smokers, 8.2 % had diabetes, and 24.6 % had hypertension. Mean CAC scores were 17.8 ± 81.8 at baseline and 29.2 ± 114.9 at follow-up. Study participants in the highest baPWV quartiles were more likely to be older, male, or heavy drinkers and have diabetes or hypertension; higher blood pressure, glucose, total cholesterol, triglyceride, ALT, GTP, and CRP; and lower HDL (Table 1).

**Table 1:** Baseline characteristics of study participants by brachial-ankle pulse wave velocity quartiles

Characteristics	PWV quartiles				P for trend
	Q1	Q2	Q3	Q4	
PWV (cm/sec)	977–1256.5	1256.5–1345.5	1345.5–1441.5	1441.5–2397.5	<0.001
Number	281	281	282	280	
Age (years)^a^	42.3 (4.5)	43.3 (4.3)	43.8 (5.2)	45.0 (6.0)	<0.001
Male (%)	89.7	94.0	96.8	99.3	0.001
BMI (kg/m^2^)	25.1(3.3)	24.9 (2.8)	25.1 (3.2)	25.4 (2.9)	0.155
Obesity (%)	43.8	47.1	53.6	52.9	0.012
Current smoker (%)	26.3	31.0	30.9	33.9	0.065
Alcohol intake (%)^b^	28.1	32.4	32.3	40.7	0.003
High education level (%)^c^	84.2	82.3	87.7	80.8	0.655
Diabetes (%)	5.0	6.4	9.6	16.1	<0.001
Hypertension (%)	10.3	23.8	26.2	42.9	<0.001
Systolic BP (mmHg)^a^	113.9 (10.8)	118.7 (11.5)	119.3 (11.2)	126.5 (11.5)	<0.001
Diastolic BP (mmHg)^a^	72.6 (8.1)	76.0 (8.8)	76.5 (8.0)	81.5 (9.0)	<0.001
Glucose (mg/dL)^a^	94.4 (11.1)	98.1 (18.3)	99.2 (18.1)	105.3 (23.6)	<0.001
Total cholesterol (mg/dL)^a^	203.5 (37.4)	212.0 (35.5)	213.6 (39.5)	210.8 (37.5)	0.020
LDL-C (mg/dL)^a^	126.4 (34.1)	133.5 (32.3)	134.6 (34.6)	130.1 (33.8)	0.174
HDL-C (mg/dL)^a^	53.2 (13.8)	50.5 (10.7)	49.7 (11.4)	51.0 (12.0)	0.022
Triglycerides (mg/dL)^d^	114 (80–156)	138 (98–192)	139 (99–200)	163 (114–227.5)	<0.001
ALT (U/l)^d^	22 (16–31)	24 (18–35)	27 (20–41)	28 (20–42)	<0.001
GTP (U/l)^d^	29 (19–43)	35 (24–54)	40 (26–58)	41 (28.5–68)	<0.001
C-reactive protein (mg/L)^d^	0.05 (0.03–0.12)	0.06 (0.04–0.11)	0.07 (0.04–0.11)	0.07 (0.04–0.14)	0.005

Data are ^a^ means (standard deviation), ^d^ medians (interquartile range), or percentages

SI unit conversion (multiply the conversion factors to obtain the SI unit): glucose, 0.0555 (mmol/L); Total cholesterol, 0.0259 (mmol/L); LDL-C, 0.0259 (mmol/L); HDL-C, 0.0259 (mmol/L); Triglyceride, 0.0113 (mmol/L); C-reactive protein, 10 (mg/L)

*ALT* alanine aminotransferase, *GTP* glutamyl transpeptidase, *BMI* body mass index, *BP* blood pressure, *HDL-C* high-density lipoprotein-cholesterol, ^b^ ≥20 g/day; ^c^ ≥College graduate

During the median 2.7-year follow-up period, 318 subjects (28.3 %) had an increased CAC score at follow-up examination compared with baseline. CAC progression was seen in 76 of 835 subjects (9.1 %) with baseline CAC = 0 but in 242 of 289 subjects (83.7 %) with baseline CAC > 0. Compared with subjects with no change in CAC values, those with an increase in CAC levels were more likely to be older, male, obese, or heavy drinkers and have diabetes or hypertension; higher BMI, blood pressure, fasting glucose, LDL, ALT, GTP, or CRP; and lower HDL cholesterol (Table 2). Study participants in the highest baPWV quartiles were more likely to have more severe progression of CAC compared with those in the lower baPWV quartiles (Table 3).

**Table 2:** Baseline characteristics of study participants by Coronary Calcium Score change in serial examination

Characteristics	CAC change		P for trend
	CAC change = 0	CAC change >0	
Number	806	318	
Age (years)^a^	42.9 (5.0)	45.3 (5.2)	<0.001
Male (%)	93.1	99.7	<0.001
BMI (kg/m^2^)	24.9 (3.0)	25.8 (2.9)	<0.001
Obesity (%)	45.4	59.1	<0.001
Current smoker (%)	32.5	30.9	0.605
Alcohol intake (%)^b^	32.3	38.7	0.018
High education level (%)^c^	82.9	86.2	0.188
Diabetes (%)	7.1	14.5	<0.001
Hypertension (%)	21.8	35.9	<0.001
Systolic BP (mmHg)^a^	118.9 (12.3)	121.3 (11.4)	0.003
Diastolic BP (mmHg)^a^	76.1 (9.1)	77.9 (8.7)	0.002
Glucose (mg/dL)^a^	97.6 (16.4)	103.4 (23.2)	<0.001
Total cholesterol (mg/dL)^a^	206.4 (37.1)	219.1 (37.6)	<0.001
LDL-C (mg/dL)^a^	127.9 (33.0)	139.5 (34.5)	<0.001
HDL-C (mg/dL)^a^	51.7 (12.4)	49.6 (11.0)	<0.001
Triglycerides (mg/dL)^d^	130 (90–191)	153 (113–215)	<0.010
ALT (U/l)^d^	25 (18–37)	28 (20–40)	<0.001
GTP (U/l)^d^	34 (22–54)	40 (27–65)	<0.001
C-reactive protein (mg/L)^d^	0.06 (0.03–0.11)	0.07 (0.04–0.13)	0.022

Data are ^a^means (standard deviation), ^d^medians (interquartile range), or percentages

SI unit conversion (multiply the conversion factors to obtain the SI unit): glucose, 0.0555 (mmol/L); Total cholesterol, 0.0259 (mmol/L); LDL-C, 0.0259 (mmol/L); HDL-C, 0.0259 (mmol/L); Triglyceride, 0.0113 (mmol/L); C-reactive protein, 10 (mg/L)

*ALT* alanine aminotransferase, *GTP* glutamyl transpeptidase, *BMI* body mass index, *BP* blood pressure, *HDL-C* high-density lipoprotein-cholesterol; ^b^ ≥20 g/day; ^c^ ≥College graduate

**Table 3:** Degree of change in Coronary Calcium Score according to brachial-ankle pulse wave velocity quartiles

Characteristics	PWV quartiles				P for trend
	Q1 (N = 281)	Q2 (N = 281)	Q3 (N = 282)	Q4 (N = 280)	
Amount of interval change					<0.001
No interval change	232	219	184	171	
1–9	117	19	33	31	
10–99	29	38	59	60	
≥100	3	5	6	18	

In multivariate logistic regression analysis, baseline PWV was significant correlated with CAC progression during follow-up, especially among subjects in the third and fourth quartiles (adjusted OR, 2.04; 95 % CI 1.33–3.15 and OR, 2.14; 95 % CI 1.34–3.41, respectively) compared with those in the lowest quartile (P for trend <0.001) (Table 4). In diabetic patients, these associations remained significant for subjects in the third and fourth quartiles (adjusted OR, 3.50; 95 % CI 1.80–5.20 and OR 3.85; 95 % CI 1.88–5.82, respectively). Another analyses using SQRT method to determine CAC progression and baPWV also showed significant correlation between them (Additional file 1: Tables 1, 2). Using a cutoff point of 10 in CAC change, the progression of CAC was still significantly associated with baseline baPWV (Additional file 1: Table 3).

**Table 4:** The risk of progression of Coronary Calcium Score according to baseline brachial-ankle pulse wave velocity quartiles odds ratio (95 % CI) of CAC change >0 by PWV quartiles

	Number	Cases	Age-adjusted OR^a^ (95 % CI)	Multivariate-adjusted OR^a^ (95 % CI)	
				Model 1	Model 2
Total					
Q1	281	47	1.00 (reference)	1.00 (reference)	1.00 (reference)
Q2	281	63	1.29 (0.84–1.98)	1.12 (0.72–1.75)	1.16 (0.74–1.82)
Q3	282	99	2.31 (1.54–3.47)	1.95 (1.27–2.99)	2.04 (1.33–3.15)
Q4	280	109	2.34 (1.55–3.52)	1.90 (1.22–2.95)	2.14 (1.34–3.41)
P for trend			<0.001	0.001	<0.001

*OR* odds ratio, *CI* confidence interval

^a^Estimated from logistic regression. Multivariable model 1 was adjusted for age, sex, center, year of screening exam, smoking status, alcohol intake, educational level, BMI, diabetes, hypertension, HDL, LDL and glucose; model 2: model 1 plus adjustment for sbp and heart rate

Among study subjects with baseline CAC = 0, higher baPWV could better predict the likelihood of CAC progression compared with lower baPWV quartile values (Table 5), although baseline PP could not predict CAC progression (higher PP group, adjusted hazard ratio [HR], 0.92; 95 % CI 0.63–1.27, compared with lower half of PP). After analyzing the interaction between PP and PWV, PP was still not a significant predictor or CAC progression. Only higher PWV (>50 %), regardless of PP, was significantly associated with CAC progression (Table 6).

**Table 5:** Risk of progression of Coronary Calcium Score according to baseline brachial-ankle pulse wave velocity quartiles odds ratio (95 % CI) of CAC change >0 by PWV quartiles among subjects with baseline CAC = 0

	Number	Cases	Age-sex-adjusted OR^a^ (95 % CI)	Multivariate-adjusted OR^a^ (95 % CI)	
				Model 1	Model 2
Total					
Q1	236	8	1.00 (reference)	1.00 (reference)	1.00 (reference)
Q2	219	17	1.22 (0.93–5.27)	2.28 (0.94–5.51)	1.26 (0.93–5.50)
Q3	202	30	4.38 (1.95–9.83)	4.21 (1.83–9.68)	1.15 (1.79–9.61)
Q4	178	21	3.05 (1.31–7.12)	3.16 (1.29–7.70)	3.04 (1.20–7.73)
P for trend			0.003	0.004	0.006

*OR* odds ratio, *CI* confidence interval

^a^Estimated from logistic regression. Multivariable model 1 was adjusted for age, sex, center, year of screening exam, smoking status, alcohol intake, educational level, BMI, diabetes, hypertension, HDL, LDL and glucose; model 2: model 1 plus adjustment for sbp and heart rate

**Table 6:** Risk of progression of Coronary Calcium Score according to pulse pressure and brachial-ankle pulse wave velocity odds ratio^a^ (95 % CI) of CAC change >0 by PWV50 % and pulse pressure 50 %

	Number	Cases	Age and sex-adjusted OR^a^ (95 % CI)	Multivariate-adjusted OR^a^ (95 % CI)	
				Model 1	Model 2
Pulse pressure					
Pulse pressure <50 %	574	159	1.00 (reference)	1.00 (reference)	1.00 (reference)
Pulse pressure ≥50 %	550	159	1.01 (0.77–1.32)	0.91 (0.68–1.21)	0.90 (0.63–1.28)
Pulse Pressure and Pulse Wave Velocity					
PWV <50 % and pulse pressure <50 %	322	66	1.00 (reference)	1.00 (reference)	1.00 (reference)
PWV <50 % and pulse pressure ≥50 %	240	44	0.90 (0.58–1.39)	0.85 (0.54–1.33)	0.90 (0.55–1.46)
PWV ≥50 % and pulse pressure <50 %	252	93	2.01 (1.37–2.95)	1.82 (1.22–2.72)	1.88 (1.26–2.82)
PWV ≥50 % and pulse pressure ≥50 %	310	115	1.88 (1.30–2.72)	1.57 (1.06–2.32)	1.75 (1.09–2.80)
P for trend			<0.001	0.002	<0.001

*OR* odds ratio, *CI* confidence interval

^a^Estimated from logistic regression. Multivariable model 1 was adjusted for age, sex, center, year of screening exam, smoking status, alcohol intake, educational level, BMI, diabetes, hypertension, HDL, LDL and glucose; model 2: model 1 plus adjustment for sbp and heart rate

In multivariate analysis, progression to CAC > 0 among 835 subjects with CAC = 0 was associated with male sex (OR 3.10; 95 % CI 2.25–3.95; P < 0.001), diabetes (OR 1.90; 95 % CI 1.05–2.85; P = 0.004), and higher PWV (OR 1.85; 95 % CI 1.20–2.45; P = 0.001). However, among the 289 individuals with baseline CAC > 0, only the presence of CAC itself (OR, 14.85; 95 % CI 12.10–17.25; P < 0.001) rather than other CAD risk factors was predictive of CAC progression.

## Discussion

Our study clearly demonstrates that arterial stiffness, measured by baPWV, can predict progression of CAC in Korean adults and suggests that noninvasive, nonradiating, and readily available baPWV could be a better surrogate marker of CAC progression than PP. The findings of our study are consistent with those of other studies showing a significant association between several surrogate markers of arterial stiffness (measured using various parameters) and CAC [14, 16, 17, 21, 22].

Several clinical risk factors including microalbuminuria or statin use are related to CAC incidence and progression, especially in patients with diabetes mellitus [23–26]. CAC nearly inevitably progress, with limited influence of traditional risk factors, so prediction or calculation of progression of CAC might be important [25, 27]. Because CAC scanning using MDCT is a test commonly used in clinical practice as a single examination rather than serial examinations, performance of multiple CAC tests in the same setting might be impracticable and hazardous owing to radiation exposure. Although serial measurements of CAC might be meaningful, no intervention, including statin therapy [28], has been shown to slow CAC progression, and serial measurements of CAC are of unproven clinical value. On the other hand, baPWV is a relatively simple, noninvasive, nonradiating, and readily available combined measure of central and peripheral arterial stiffness. Unlike carotid–femoral pulse wave velocity, which is a measure of central arterial stiffness, baPWV is a combined measure of central and peripheral arterial stiffness. BaPWV may provide information similar to that derived from central PWV [15] and is independently associated with the presence and severity of CAC [16, 17, 29]. Our study shows a significant correlation between baPWV and CAC progression in apparently healthy subjects. From a clinical perspective, this convenient, nonradiating, and easily available tool may help identify CAC progression, irrespective of CAC presence.

Several plausible mechanisms may be responsible for the association between baPWV and coronary atherosclerosis as expressed by CAC. First, some common risk factors possibly contribute to arterial stiffness and atherosclerosis, and this is more likely in the peripheral arteries. ABI is also associated with the incidence and severity of coronary atherosclerosis [30]. Secondly, given that the aorta forms a large portion of the arterial tree over which baPWV is measured, 58 % of the variation in baPWV can be explained by aortic PWV [15]. Thus, baPWV is not only a marker of peripheral arterial stiffness but also an indirect marker of central arterial stiffness. Thirdly, arterial stiffness could increase the mechanical shear stress on the arterial wall side and cardiac afterload. This can trigger the initial pathophysiologic cascade, which leads to atherosclerotic changes and cardiac remodeling.

Diabetes mellitus is associated with an increased risk of arterial stiffness and atherosclerotic cardiovascular disease. [31] Vascular calcification, including coronary artery calcification, significantly improved the prediction of outcome compared with the consideration of traditional risk factors, [32, 33] and baPWV improved the ability to identify diabetic individuals at high risk of future cardiovascular events. [34] The combined assessment of CAC and baPWV also could more effectively predict cardiac events than the CAC score alone [35]. Therefore, to predict future events, baPWV might have the potential for wide clinical applications, especially in diabetic patients.

Our study showed that PP, a surrogate marker of arterial stiffness, could not predict CAC progression, unlike baPWV. Generally, increases in PP result from factors that increase and/or decrease systolic and diastolic pressure, respectively. Changes in PP are mostly related to changes in systolic blood pressure, particularly among the elderly, and are usually the result of stiffness in the large arteries as well as an early pulse wave reflection. Although increases in peripheral vascular resistance appear to be a relatively more important component of hypertension in younger patients (younger than 50 years), the role of peripheral vascular resistance in hypertension diminishes progressively with age [36, 37]. Given the known effects of aging on vessel stiffness, increases in PP in older patients result from aortic stiffening, whereas increases in younger patients are more likely to result from increases in stroke volume. Subjects in our cohort had an average age of 43.6 years, which means that higher PP is not likely related to higher arterial stiffness and as a result is not associated with CAC progression.

In our study, during the follow-up period of 2.7 ± 0.5 years, approximately 9.1 % of subjects (76/835) with CAC = 0 at baseline had increases in CAC, whereas CAC progression was observed in 83.7 % of subjects (242/289) with a baseline CAC > 0. This conversion rate is very similar to that reported in a previous study [38]. In that study, 106 of 422 subjects (25.1 %) with no baseline CAC had a positive CAC score during the follow-up period. Conversion from a CAC score of 0 to >0 occurred in 2 (0.5 %), 5 (1.2 %), 24 (5.7 %), 26 (6.2 %), and 49 (11.6 %) subjects after 1, 2, 3, 4, and 5 years of follow up, respectively [38]. Our study also showed that the presence of diabetes, male sex, and higher baPWV were associated with CAC progression in subjects with baseline CAC = 0 whereas in subjects with baseline CAC > 0, only the presence of CAC could predict CAC progression. So far, no clinical factor seems to mandate repeat CAC scanning, but baPWV is able to help predict CAC progression.

### Limitations

The chief limitation of our study was a lack of data regarding how patients with normal CAC values were treated by their physicians. The treatment of risk factors such as hypertension, diabetes, or dyslipidemia was left to the discretion of the physicians and subjects, which could have resulted in significant treatment bias. Thus, it is difficult to determine the magnitude of any confounding effects of treatment for risk factors that may have altered the natural course of CAC progression. Additional unmeasured confounders that can modify calcification processes, such as fetuin A, novel inflammatory markers, and additional risk factors (e.g., renal failure, thyroid status, or family history), may mediate this accelerated risk. This study did not evaluate such markers but instead limited its evaluation to the association between baPWV and CAC progression. Secondly, the study design was cross-sectional, which might preclude casual correlation. Thirdly, arterial stiffness was measured using baPWV, for which there is a relative lack of scientific evidence compared with carotid-femoral PWV, the gold standard of arterial stiffness measurement. However, previous studies have shown a high correlation between baPWV and aortic PWV [39]. Currently, baPWV is widely used in Asia, and evidence of its value is growing with time. Fourthly, our study comprised Korean adults voluntarily attending a health-screening program and was primarily performed in men, a small proportion of whom had diabetes. Fifthly, a CAC score between 0 and 10 is likely to represent noise. Although the reproducibility of CAC scores is high, the reproducibility of scores between 0 and 9 is an issue. As compared to those who scored 10 or greater, variation was very high among subjects who scored between 0 and 9, which might have had a significant impact on our results. Finally, our follow-up period was relatively short, without evaluation of clinical outcomes, which gives our findings limited generalizability.

## Conclusions

Higher arterial stiffness measured by baPWV could be significantly associated with CAC progression.

## Additional file

10.1186/s12933-015-0311-3 **Table 1.** The Risk of Progression of Coronary Calcium Score According to Baseline Brachial-Ankle Pulse Wave Velocity: Odds Ratio (95 % Confidence Interval) of Difference [√CAC_(follow-up)_ − √CAC_(baseline)_] >2.5 by Pulse Wave Velocity quartiles—The SQRT Analysis. **Table 2.** The Risk of Progression of Coronary Calcium Score According to Pulse Pressure and Brachial-ankle Pulse Wave Velocity: Odds ratio (95 % Confidence Interval) of Difference [√CAC_(follow-up)_ − √CAC_(baseline)_] >2.5 by Pulse Wave Velocity 50 % and pulse pressure 50 %—The SQRT Analysis. **Table 3.** The Risk of Progression of Coronary Calcium Score According to Baseline Brachial-Ankle Pulse Wave Velocity: Odds ratio (95 % Confidence Interval) of Coronary Calcium Score Change change >=10 by Pulse Wave Velocity Quartiles.

## Abbreviations

ABI: ankle-brachial index
ALT: alanine transaminase
baPWV: brachial-ankle pulse wave velocity
BMI: body-mass index
CAC: coronary artery calcium
CAD: coronary artery disease
CI: confidence interval
CT: computed tomography
GTP: gamma-glutamyltransferase
hsCRP: high-sensitivity C-reactive protein
HDL: high-density lipoprotein
LDL: low-density lipoprotein
MDCT: multidetector computed tomography
OR: odds ratio
PP: pulse pressure
PWV: pulse wave velocity
