# Supplementary material for: Association between brachial-ankle pulse wave velocity and progression of coronary artery calcium: a prospective cohort study
Source: Cardiovasc Diabetol. 2015 Nov 4;14:147. doi: 10.1186/s12933-015-0311-3 (PMC4632351; doi:10.1186/s12933-015-0311-3)
Supplement: Supplementary file 1 — 10.1186/s12933-015-0311-3 Table 1. The Risk of Progression of Coronary Calcium Score According to Baseline Brachial-Ankle Pulse Wave Velocity: Odds Ratio (95 % Confidence Interval) of Difference [√CAC(follow-up) − √CAC(baseline)] >2.5 by Pulse Wave Velocity quartiles—The SQRT Analysis. Table 2. The Risk of Progression of Coronary Calcium Score According to Pulse Pressure and Brachial-ankle Pulse Wave Velocity: Odds ratio (95 % Confidence Interval) of Difference [√CAC(follow-up) − √CAC(baseline)] >2.5 by Pulse Wave Velocity 50 % and pulse pressure 50 %—The SQRT Analysis. Table 3. The Risk of Progression of Coronary Calcium Score According to Baseline Brachial-Ankle Pulse Wave Velocity: Odds ratio (95 % Confidence Interval) of Coronary Calcium Score Change change >=10 by Pulse Wave Velocity Quartiles. [file 12933_2015_311_MOESM1_ESM.docx]

**Additional Materials**

**Table 1. The Risk of Progression of Coronary Calcium Score According to Baseline Brachial-Ankle Pulse Wave Velocity Quartiles**

**odds ratio^a^ (95% CI) of difference [√CAC_(follow-up)_ – √CAC_(baseline)_] >2.5 by PWV quartiles - The SQRT Analysis**

|  | Number | Cases | Age-sex -adjusted OR^a^ (95% CI) | Multivariate-adjusted OR^a^  (95% CI) | |  |
| --- | --- | --- | --- | --- | --- | --- |
|  |  |  |  | Model 1 | Model 2 | |
| Total |  |  |  |  |  | |
| Q1 | 281 | 17 | 1.00 (reference) | 1.00 (reference) | 1.00 (reference) | |
| Q2 | 281 | 27 | 1.51 (0.80-2.85) | 1.26 (0.66-2.42) | 1.33 (0.69-2.55) | |
| Q3 | 282 | 45 | 2.57 (1.43-4.64) | 2.04 (1.11-3.75) | 2.19 (1.18-4.04) | |
| Q4 | 280 | 56 | 3.01 (1.68-5.38) | 2.32 (1.26-4.28) | 2.76 (1.45-5.27) | |
| P for trend |  |  | <0.001 | 0.002 | <0.001 | |

^a^ Estimated from logistic regression. Multivariable model 1 was adjusted for age, sex, center, year of screening exam, smoking status, alcohol intake, educational level, BMI, diabetes, hypertension, HDL, LDL and glucose; model 2: model 1 plus adjustment for sbp and heart rate

**Table 2. The Risk of Progression of Coronary Calcium Score According to Pulse Pressure and Brachial-ankle Pulse Wave Velocity odds ratio^a^ (95% CI) of difference [√CAC_(follow-up)_ – √CAC_(baseline)_] >2.5 by PWV50% & pulse pressure 50% - The SQRT Analysis**

|  | Number | | Cases | Age sex-adjusted OR^a^ (95% CI) | Multivariate-adjusted OR^a^ (95% CI) | |
| --- | --- | --- | --- | --- | --- | --- |
|  |  |  |  |  | Model 1 | Model 2 |
| Pulse pressure |  | |  |  |  |  |
| Pulse pressure <50% | | 574 | 81 | 1.00 (reference) | 1.00 (reference) | 1.00 (reference) |
| Pulse pressure ≥50% | | 550 | 64 | 0.76 (0.53-1.09) | 0.69 (0.47-1.00) | 0.60 (0.38-0.95) |
| Pulse Pressure and Pulse Wave Velocity | | | |  |  |  |
| PWV <50% & pulse pressure <50% | | 322 | 33 | 1.00 (reference) | 1.00 (reference) | 1.00 (reference) |
| PWV <50% & pulse pressure ≥50% | | 240 | 11 | 0.43 (0.21-0.87) | 0.40 (0.20-0.83) | 0.38 (0.19-0.82) |
| PWV ≥50% & pulse pressure <50% | | 252 | 48 | 1.83 (1.13-2.97) | 1.56 (0.95-2.58) | 1.60 (0.96-2.66) |
| PWV ≥50% & pulse pressure ≥50% | | 310 | 53 | 1.52 (0.94-2.44) | 1.22 (0.74-2.02) | 1.18 (0.64-2.16) |
| P for trend | |  |  | 0.005 | 0.065 | 0.022 |

^a^ Estimated from logistic regression. Multivariable model 1 was adjusted for age, sex, center, year of screening exam, smoking status, alcohol intake, educational level, BMI, diabetes, hypertension, HDL, LDL and glucose; model 2: model 1 plus adjustment for sbp and heart rate

**Table 3. The Risk of Progression of Coronary Calcium Score According to Baseline Brachial-Ankle Pulse Wave Velocity Quartiles**

**odds ratio^a^ (95% CI) of CAC change >=10 by PWV quartiles**

|  | Number | Cases | Age-sex -adjusted OR^a^ (95% CI) | Multivariate-adjusted OR^a^  (95% CI) | |  |
| --- | --- | --- | --- | --- | --- | --- |
|  |  |  |  | Model 1 | Model 2 | |
| Total |  |  |  |  |  | |
| Q1 | 281 | 32 | 1.00 (reference) | 1.00 (reference) | 1.00 (reference) | |
| Q2 | 281 | 43 | 1.27 (0.77-2.09) | 1.07 (0.64-1.80) | 1.12 (0.67-1.88) | |
| Q3 | 282 | 65 | 2.00 (1.25-3.19) | 1.61 (0.99-2.61) | 1.69 (1.03-2.77) | |
| Q4 | 280 | 78 | 2.24 (1.41-3.57) | 1.65 (1.00-2.71) | 1.90 (1.12-3.22) | |
| P for trend |  |  | <0.001 | 0.015 | 0.004 | |

^a^ Estimated from logistic regression. Multivariable model 1 was adjusted for age, sex, center, year of screening exam, smoking status, alcohol intake, educational level, BMI, diabetes, hypertension, HDL, LDL and glucose; model 2: model 1 plus adjustment for sbp and heart rate
